# Special Issue “Molecular Insights into Hair Regeneration”

**DOI:** 10.3390/ijms27010477

**Published:** 2026-01-02

**Authors:** Jong-Hyuk Sung

**Affiliations:** Epi Biotech Co., Ltd., Incheon 21983, Republic of Korea; brian99@epibiotech.com; Tel.: +82-70-4209-0556

## 1. Introduction

Hair follicle development and regeneration depend on an intricate interplay of molecular signals, genetic factors, and environmental influences. With hundreds of millions experiencing hair loss globally, there is high demand for effective therapies, yet existing treatments are limited in scope and often produce incomplete results [1,2]. To address this challenge, the *International Journal of Molecular Sciences* launched a Special Issue, entitled “Molecular Insights into Hair Regeneration,” aimed at advancing our understanding of hair growth biology and paving the way for therapeutic innovations. This Editorial synthesizes the findings of six contributions in this Special Issue—four original research articles and two comprehensive reviews—which, together, push the boundaries of hair regeneration research. These studies cover a diverse range of strategies, from novel bioactive compounds and metabolic regulators to cutting-edge biophysical and genetic therapies. Below, we highlight each article’s key findings, underscoring their novelty and translational value. Then, we present some concluding remarks on their broader implications for hair biology and future therapies.

## 2. An Overview of Published Articles

### 2.1. Iris-Derived Exosomes Combat Androgenic Follicular Stress

Kim et al. (contribution 1) demonstrate a novel plant-based approach to protecting hair follicles from androgenic stress, using exosomes derived from *Iris germanica* L. rhizomes. In human dermal papilla cell cultures, these Iris-derived exosomes were found to have significantly attenuated dihydrotestosterone (DHT)-induced damage by reducing oxidative stress, thus lowering the concentrations of intracellular reactive oxygen species and restoring mitochondrial function, and thereby preventing the cellular dysfunction that leads to follicle degeneration. The exosome treatment also enhanced pro-regenerative cellular processes, including increased dermal papilla cell migration, elevated alkaline phosphatase activity, and the robust formation of 3D spheroids that mimic the hair follicle microenvironment. Notably, Iris-derived exosomes activated the canonical Wnt/β-catenin signaling pathway in the damaged cells, stabilizing β-catenin and promoting its nuclear translocation, which in turn upregulated genes essential for hair growth. This study is the first to report the therapeutic potential of plant-derived exosomes in an alopecia context. By mitigating androgen-induced oxidative injury and bolstering a key hair growth pathway, Iris-derived exosomes emerge as a promising bioactive tool for hair loss treatment. The translational implications are significant: plant nanovesicles could be developed as safe, natural ingredients in future hair loss therapeutics or cosmeceuticals, representing a novel mechanism with which to support follicle regeneration.

### 2.2. Metabolic Modulation of Anagen via Fatty Acids

Pan et al. (contribution 2) investigate hair cycle regulation from a metabolic perspective, conducting research that examines whether altering cellular energy pathways can trigger the anagen (growth) phase of hair follicles. This study addresses a critical question: are metabolic cues as important as inflammatory signals in promoting hair regrowth? Using a mouse model, the authors found that the topical application of free long-chain fatty acids had a significant effect on hair cycle re-entry. In particular, applying palmitic acid or oleic acid to the skin significantly accelerated the onset of anagen, whereas the presence of lactate (a glycolytic end-product) delayed it. These results suggest that reducing oxidative phosphorylation in the follicular environment (along with palmitate or oleate treatment) can stimulate hair follicles to exit the resting phase. Crucially, the authors also assessed inflammatory responses: neither palmitic nor oleic acid induced any significant cytokine release in skin keratinocytes or fibroblasts, and only palmitic acid triggered a mild pro-inflammatory response in immune cells (monocytes), whereas oleic acid caused virtually no inflammation. After directly comparing metabolic activation with inflammation, this study concludes that metabolic regulation holds significant promise for promoting hair growth. This novel insight shifts the focus toward bioenergetic modulation as a strategy for hair regeneration. Its translational value lies in the prospect of metabolic-based therapies—for example, topical metabolic modulators or dietary-derived compounds—that could safely induce the hair growth phase without depending on broad immunosuppression or hormonal interventions.

### 2.3. Antioxidant-Rich Microalgal Oil Promotes Hair Growth

Ying et al. (contribution 3) report the hair growth benefits of a bioactive oil derived from a marine microalga, representing an innovative antioxidant-based approach to combat hair loss. The team used oil extracted and fermented from Nannochloropsis salina, and then characterized its composition and bioactivity in the context of hair follicle health. Gas chromatography–mass spectrometry (GC–MS) analysis revealed that the Nannochloropsis fermented oil (NSO) is rich in long-chain polyunsaturated and saturated fatty acids, notably eicosapentaenoic acid (EPA), palmitic acid, and linoleic acid. Functional assays showed that NSO markedly promotes the proliferation of human dermal papilla cells and protects these critical hair follicle cells from oxidative stress-induced damage. Genome-wide expression profiling further demonstrated that NSO activates hair growth-related signaling pathways and upregulates antioxidant genes in dermal papilla cells, suggesting the existence of a mechanism whereby NSO enhances the cells’ pro-growth and stress-resistance capacities. Impressively, when applied topically to mice, NSO significantly accelerated hair regrowth: treated animals exhibited longer and heavier hair fibers, an increased number of hair follicles, greater follicle depth, and a thicker dermal layer compared to controls. By effectively stimulating hair growth and mitigating oxidative damage, this microalgal extract demonstrates considerable translational potential. It introduces a naturally derived, antioxidant-rich therapy that could be developed into a topical treatment or nutraceutical for hair loss prevention and hair regeneration. Importantly, NSO’s efficacy, combined with its likely low toxicity, positions it as a compelling candidate for further clinical research in alopecia management.

### 2.4. Radiofrequency Stimulation for Follicle Regeneration

Martínez-Pascual et al. (contribution 4) provide pioneering evidence that biophysical stimulation using radiofrequency (RF) energy can rejuvenate hair follicles, representing a potential non-pharmacological therapy for androgenetic alopecia. While RF-based devices have been utilized in clinical settings for hair loss, their effects at the cellular and molecular levels were not well understood. In this study, hair follicle explants from patients with androgenetic alopecia were treated ex vivo with a capacitive resistive electrical transfer (CRET) device delivering intermittent 448 kHz RF currents under non-thermal conditions. The RF treatment produced several pro-regenerative changes in the balding follicles: it significantly increased cell proliferation (as displayed by the higher Ki-67 labeling of follicle cells) and decreased apoptosis (demonstrated by TUNEL assays indicating reduced cell death). Additionally, RF exposure enhanced follicular differentiation signals—notably increasing β-catenin activity in hair matrix cells—and preserved tissue integrity by maintaining collagen structure and limiting matrix metalloproteinase (MMP9) expression. Strikingly, treated follicles exhibited an expansion of the stem cell-rich bulge region: the number of melanoblasts (pigment-forming precursors) in the bulge increased, and the epidermis surrounding the follicle thickened, indicative of a healthier microenvironment for hair growth. These multifaceted improvements strongly support the efficacy of RF stimulation in reversing key aspects of follicle miniaturization. From a translational standpoint, the work suggests that CRET/RF therapy could be developed as a safe, drug-free intervention to promote hair follicle regeneration. The authors rightly remark that clinical trials are necessary to confirm these promising effects in vivo, but their findings lay a mechanistic foundation for the creation of RF-based alopecia treatments and encourage even more bioengineering innovations in this arena.

### 2.5. Mechanistic Insights and Treatments for Androgenetic Alopecia

Sekhavat et al. (contribution 5) contribute a broad review that synthesizes current knowledge of androgenetic alopecia (AGA) pathophysiology and leverages these insights to survey advanced treatment strategies. AGA—the most common form of hair loss in men and women—is characterized by the progressive miniaturization of hair follicles under the influence of androgens and genetic predispositions. The review outlines how dihydrotestosterone (DHT) and other factors disrupt the crosstalk between dermal papilla cells and the follicular epithelium, leading to shortened hair growth cycles and follicle shrinking. Importantly, the authors underscore the limitations of existing approved therapies: although topical minoxidil and oral finasteride are somewhat effective, they must be used indefinitely and can cause side effects. In light of these challenges, many emerging therapies aim to more directly target the molecular drivers of AGA. They review a range of such interventions—including novel anti-androgens, enzyme inhibitors, growth factor modulators, immunomodulatory agents, and cell-based therapies—that have shown potential to promote hair regrowth by intervening in the AGA disease process. Notably, the review highlights the advantage of combination therapies: using multiple agents in concert can address different pathogenic mechanisms simultaneously, often leading to better outcomes than single-drug treatments. This emphasis on multi-targeted regimens is a key translational insight from the review. It suggests that the future of AGA management may lie in personalized combination therapies that are informed by an individual’s specific molecular pathology.

### 2.6. Advances in siRNA Delivery for Hair Loss Therapy

Jin and Sung (contribution 6) shift the focus toward next-generation molecular therapeutics for hair loss—specifically, the use of small interfering RNA (siRNA) to selectively silence pathogenic genes—and discuss the ways we can overcome the delivery challenges that have limited this approach. While siRNA-based therapy holds great promise for conditions like alopecia (due to its ability to knock down target genes involved in hair follicle degeneration), free siRNA molecules face numerous barriers: they are unstable in the bloodstream, are poorly taken up by cells, and can elicit off-target effects or immune responses. This review provides a detailed overview of innovative siRNA delivery platforms designed to address these challenges. The authors describe a variety of carrier systems—including lipid nanoparticles, polymer conjugates, dendrimers, and cell-penetrating peptides—that have been engineered to protect siRNA, enhance its uptake by hair follicle cells, and ensure that its activity is sustained in the scalp environment. They also examine current progress in the field by analyzing recent clinical trials of hair loss treatments and drawing lessons from the use of siRNA drugs approved for other diseases. Based on these analyses, Jin and Sung outline strategic design principles for clinically translatable siRNA therapies targeting hair loss, emphasizing the need for tunable delivery systems that are tailorable to the specific, unique conditions of the hair follicle niche. The translational significance of this review is clear: it bridges cutting-edge RNA interference technology with practical considerations for real-world hair loss treatment. By drawing attention to successful delivery strategies and proposing frameworks for future research, the article lays the groundwork for the development of safe and effective gene-silencing interventions as a new class of therapeutics for alopecia.

## 3. Concluding Remarks

Collectively, the articles in this Special Issue illustrate the vibrant progress and multifaceted nature of modern hair regeneration research. From plant-derived exosomes and marine oils to metabolic modulation, bioelectric stimulation, and gene therapy, the studies synthesized in this collection showcase how leveraging molecular insights can lead us toward yielding novel strategies to promote hair growth. A common theme is the translational orientation of these investigations: each offers not only a more thorough understanding of hair follicle biology but also a tangible step toward innovative therapies.

We encourage readers to engage with the full content of these contributions, as, together, they highlight emerging opportunities to address and overcome the longstanding challenges of hair loss. The broader implication for hair biology is that solutions will likely emerge from an interdisciplinary convergence of approaches, as evidenced by this Special Issue’s diversity of topics. By integrating natural product research, metabolic biology, bioengineering, and molecular medicine, this field is moving closer to truly effective and personalized hair restoration methods. It is our hope that the advances highlighted herein will inspire further research and collaboration, ultimately accelerating the translation of these molecular insights into groundbreaking therapies for alopecia and beyond.
